# Barcoding rotifer biodiversity in Mediterranean ponds using diapausing egg banks

**DOI:** 10.1002/ece3.2986

**Published:** 2017-05-27

**Authors:** Emilio Moreno, José M. Conde‐Porcuna, Africa Gómez

**Affiliations:** ^1^ Institute of Water ResearchUniversity of GranadaGranadaSpain; ^2^ Department of EcologyFaculty of SciencesGranadaSpain; ^3^ School of Environmental SciencesUniversity of HullHullUK

**Keywords:** biodiversity, diapausing egg bank, DNA barcoding, rotifers

## Abstract

The biodiversity of Mediterranean freshwater bodies is among the most threatened worldwide; therefore, its accurate estimation is an urgent issue. However, traditional methods are likely to underestimate freshwater zooplankton biodiversity due to its high species seasonality and cryptic diversity. We test the value of applying DNA barcoding to diapausing egg banks, in combination with the creation of a reference collection of DNA barcodes using adult individual samples, to characterize rotifer communities. We use monogonont rotifers from two lakes in Doñana National Park and one from Ruidera Natural Park in Spain as models to create a reference collection of DNA barcodes for taxonomically diagnosed adult individuals sampled from the water column, to compare with the sequences obtained from individual eggs from the diapausing egg banks. We apply two different approaches to carry out DNA taxonomy analyses, the generalized mixed Yule coalescent method (GMYC) and the Automatic Barcode Gap Discovery (ABGD), to the obtained sequences and to publicly available rotifer sequences. We obtained a total of 210 new rotifer COI sequences from all three locations (151 diapausing eggs and 59 adults). Both GMYC and ABGD generated the same 35 operational taxonomic units (OTUs), revealing four potential cryptic species. Most sequences obtained from diapausing eggs (85%) clustered with sequences obtained from morphologically diagnosed adults. Our approach, based on a single sediment sample, retrieved estimates of rotifer biodiversity higher than or similar to those of previous studies based on a number of seasonal samples. This study shows that DNA barcoding of diapausing egg banks is an effective aid to characterize rotifer diversity in Mediterranean freshwater bodies.

## 1 Introduction

Mediterranean freshwater bodies are important reservoirs of biodiversity for aquatic invertebrates. They are among the most vulnerable and threatened habitats worldwide due to drastic changes in hydrological regime patterns and the introduction and natural invasions of exotic species (Briski Cristescu, Bailey, & MacIsaac, 2011; Mergeay, Verschuren, & De Meester, 2005; Myers, Mittermeier, Mittermeier, Da Fonseca, & Kent, 2000; Oertli, Biggs, Céréghino, & Grillas, 2005; Sala et al., 2000). Under this scenario, changes in the zooplankton community structure and composition can be noticeably dramatic, especially for passively dispersed species, highlighting the urgency of biodiversity assessments, which are challenging due to the time and cost of surveys. The application of conservation approaches usually fails as lakes and ponds are isolated islandlike habitats with high seasonal species turnover (Dudgeon et al., 2006). In addition, estimates of zooplankton diversity are often incomplete due to the large number of cryptic and undescribed species involved (Esteban & Finlay, 2010). Finally, freshwater biodiversity studies have often disregarded invertebrate species, which are essential for maintaining the natural dynamic of ecosystems (Muñoz, 2010).

Rotifera is one of the main groups of zooplankton and plays a key role as component of food webs in aquatic ecosystems, transferring energy from low to higher trophic levels (Wallace, 2002). The phylum is classified into three classes, Seisonidea, Bdelloidea, and Monogononta, and comprises over 2,000 described species, mostly microscopic. Monogononta, the most abundant and morphologically diverse class, includes 1,570 species (Segers, 2007) of globally distributed, short‐lived organisms found in a wide range of continental water bodies from hypersaline to freshwater. However, their supposed ubiquity has been rejected because they have shown high levels of cryptic diversity. For example, previous studies focused on rotifer alpha diversity and biogeography showed seven putative species of *Brachionus plicatilis* in Spain and 15 described species worldwide (Gómez, Serra, Carvalho, & Lunt, 2002; Mills et al., 2016), six lineages of *Brachionus calyciflorus* from China and at least eight putative species from different countries (Schröder & Walsh, 2007; Xiang et al., 2011), six putative species of *Testudinella clypeata* from the UK (Leasi, Tang, De Smet, & Fontaneto, 2013), 12 putative species of *Polyarthra dolichoptera* from different locations in Italy (Obertegger, Flaim, & Fontaneto, 2014), and eight putative species of *Keratella cochlearis* from Italy (Cieplinski, Weisee, & Obertegger, 2016). In nearly 60% of the complexes described in the phylum Rotifera, at least two species of the same complex can occur (Gabaldón, Fontaneto, Carmona, Montero‐Pau, & Serra, 2016). A further challenge involved in assessing the diversity of rotifer communities is their short life cycle with a short generation time, temperature‐dependent growth rate, and embryonic development which varies from population to population (Herzig, 1983). Monogonont rotifers complete their life cycle in 5–10 days and have a generation time threshold of 2–4 days in temperate lakes (20–22°C) (Gillooly, 2000; Ricci, 2001). Seasonal species replacement occurs faster in temporary and fluctuating environments; therefore, a higher sampling effort in terms of temporal frequency is needed to obtain unbiased assessments of species composition (Serrano & Fahd, 2005; Fahd et al., 2007).

The life cycle of monogonont rotifers includes diapausing eggs able to survive unfavorable periods of harsh conditions and prevent local extinctions (Ricci, 2001; Wallace, 2002). Diapausing eggs accumulate in the sediments, where they can persist for long periods of time and establish egg banks, potentially integrating spatiotemporal patterns of community diversity, increasing generation time and decreasing population growth rate (Brendonck & De Meester, 2003; DeStasio, 1989; Gabaldón, Serra, Carmona, & Montero‐Pau, 2015; Hairston, 1996; Vandekerkhove, Declerck, Jeppesen, et al., 2005). Given these features, the study of diapausing egg banks could potentially become a cost‐effective approach for assessment of zooplankton biodiversity, as a single egg bank sample may cover a period of several years, while plankton samples are highly variable temporally, implying that several samples are required for an accurate characterization of zooplankton communities (Duggan, Green, & Shiel, 2002; Havel, Eisenbacher, & Black, 2000; May, 1986; Mergeay et al., 2005; Vandekerkhove, Declerck, Jeppesen, et al., 2005). In other planktonic invertebrates, such as cladocerans, diapausing stages or ephippia can display very species‐specific morphologies (e.g., size, shape, color, and external sculpturing or ornamentation) and they have been successfully used for species‐level identification (Pourriot & Snell, 1983; Vandekerkhove et al., 2004). In contrast, although rotifer diapausing eggs can show genus‐specific morphological features (Walsh, May, & Wallace, 2016), high intraspecific morphological variability and absence of useful diagnostic characters have precluded their use for species‐level identification (Brendonck & De Meester, 2003; Gilbert, 1974, 1995), leading to biased diversity estimates between diapausing egg diversity and species diversity (Piscia et al., 2012). An early approach to the study of diapausing egg banks for assessing zooplankton species richness is the hatching method (Brendonck & De Meester, 2003; Duggan et al., 2002; Gleason, Euliss, Hubbard, & Duffy, 2004; May, 1986; Palazzo, Bonecker, & Fernandes, 2008; Vandekerkhove, Declerck, Brendonck, Conde‐Porcuna, Jeppesen, Johansson, et al., 2005). However, this method has limitations, and the identification of appropriate hatching cues (light, temperature, salinity, oxygen concentration, or others) and the latency period (which may vary from a few days to several months) are often species‐specific and within species (Gilbert & Walsh, 2005), and potential biases introduced by bet‐hedging, where only a fraction of the eggs hatches at a given time (García‐Roger, Serra, & Carmona, 2014; Schröder, 2005; Schwartz & Hebert, 1987; Vandekerkhove, Declerck, Brendonck, Conde‐Porcuna, Jeppesen and De Meester, 2005).

A significant improvement for the analysis of rotifer diapausing egg bank biodiversity would include the development of tools for the direct species identification of diapausing eggs. An alternative to morphological identification and hatching approaches is using molecular techniques, which can be applied on individual diapausing eggs or adults. DNA barcoding and DNA taxonomy offer a complementary tool to traditional taxonomy and to quantify global biodiversity, especially for groups whose morphology is uninformative, plastic, and/or difficult to describe (Fontaneto, Flot, & Tang, 2015; Hebert, Ratnasingham, & deWaard, 2003). DNA barcoding consists of the sequencing and analysis of a DNA fragment of the mitochondrial gene COI (the cytochrome *c* oxidase subunit I) to identify species (Hebert et al., 2003). On the other hand, DNA taxonomy approaches, such as GMYC (generalized mixed Yule coalescent; Pons et al., 2006; Fujisawa & Barraclough, 2013) or Automatic Barcode Gap Discovery (ABGD; Puillandre, Lambert, Brouillet, & Achaz, 2012), are based on the analysis of variation in genetic data for delimiting species. DNA barcoding and analytical tools of DNA taxonomy have offered a very productive approach to uncover cryptic species complexes (Birky, Wolf, Maughan, Herbertson, & Henry, 2005; Fontaneto et al., 2007; Fujisawa & Barraclough, 2013; Hebert et al., 2004; Leasi et al., 2013; Puillandre et al., 2012). DNA taxonomy has indeed revealed that most rotifer taxonomic species analyzed are actually species complexes both in monogonont (Derry, Hebert, & Prepas, 2003; Leasi et al., 2013; Mills et al., 2016; Obertegger, Fontaneto, & Flaim, 2012; Obertegger et al., 2014; Schröder & Walsh, 2007; Walsh, Schröder, Wallace, & Rico‐Martínez, 2009; Xiang et al., 2011) and in bdelloids (Fontaneto, Barraclough, Chen, Ricci, & Herniou, 2008; Fontaneto, Kaya, Herniou, & Barraclough, 2009; Fontaneto et al., 2011), with 39 known species complexes identified so far in rotifers (21 monogononta and 18 bdelloidea), a number that is likely to increase (see Fontaneto, 2014 for a recent review). DNA barcoding has also been useful as a complementary tool for surveying rotifer biodiversity in larger geographic areas, revealing the presence of several potential cryptic species (García‐ Morales & Elías‐Gutiérrez, 2013), and for studies on the dispersal and transport detection of invasive species (Briski et al., 2011; Mergeay et al., 2005).

Here, we test the value of applying DNA barcoding and DNA taxonomy to diapausing egg banks from a single sediment sample to characterize zooplankton communities. To do so, we collected sediment and water‐column samples from two lakes in Doñana National Park and one from Ruidera Natural Park in Spain. We create a reference collection of DNA barcodes with the water‐column monogonont rotifers taxonomically diagnosed as models to compare to barcodes obtained from their corresponding diapausing egg from sediment samples. We then use DNA taxonomy methods to delimit species and detect potential cryptic species, and describe species complexes in our dataset in the context of all published rotifer barcodes.

## 2 Materials and Methods

### 2.1 Study sites

**Figure 1:**
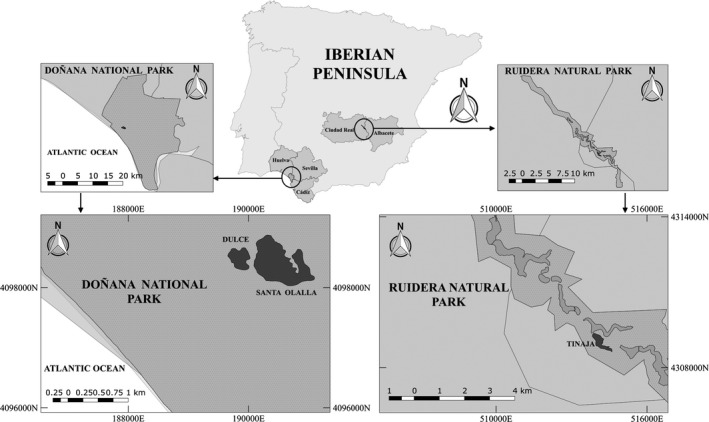
Sampling sites

Samples were taken from three lakes located in two regions with different limnological and geographic characteristics (Figure 1). Tinaja Lake is located in Ruidera Natural Park in Central Spain, between Albacete and Ciudad Real provinces. It is a warm monomictic mesotrophic and eutrophic lake, part of 15 chain‐connected lakes separated by travertine (tufa) dams and fed by groundwater and by surface drainage from upper to lower lakes when groundwater levels are high. Climate in Ruidera is continental Mediterranean, with rainfall mostly in spring and autumn. Zooplankton species composition, abundance, and biomass are variable throughout lakes, where rotifers are the most abundant taxon followed by cladoceran and copepods (Álvarez‐Cobelas et al., 2007; Bort, Rojo, Rodrigo, & Maidana, 2005; Rojo, Rodrigo, & Barón‐Rodríguez, 2007). Santa Olalla and Dulce are coastal peridunal shallow ponds located in Doñana National Park (SW Spain). The area has a Mediterranean climate with Atlantic influence that results in mild winters and dry hot summers. Rainfall is quite variable seasonally and annually, and this is reflected in drastic fluctuations of water level, physical–chemical and biological parameters, and temporal character of the freshwater bodies. Santa Olalla and Dulce are contiguous ponds fed by groundwater and rainfall, permanent and semipermanent respectively. Although both ponds are usually isolated hydrologically and have different limnological characteristics, during high‐precipitation seasons the ponds can be connected. The zooplankton composition changes seasonally and annually, with rotifers as predominant group, mostly from the genus *Brachionus*, followed by cladoceran and copepods (López, Toja, & Gabellone, 1991; Moreno, Pérez‐Martínez, & Conde‐Porcuna, 2016; Serrano & Toja, 1998).

### 2.2 Sample collection

Sediment cores of 10 cm depth were taken with a Universal Percussion Corer (diameter: 6.8 cm, length: 20 cm) from the deepest part of each lake (03/18/2009 in Doñana and 01/21/2009 in Ruidera). The first 4 cm of the core was sliced into 2‐cm sections, and the slices were packed in ziplock plastic bags, labeled, and stored at 4°C in the dark until processed. To construct a reference collection of rotifers found in the water column of the study sites, water samples were collected with a Van Dorn sampler in Ruidera or a tube sampler (6.8 cm diameter) in Doñana, filtered through a 40 μm mesh and immediately fixed in 96% ethanol (during the period 03/18/2009‐09/16/2010 in Doñana and 01/21/2009‐02/10/2010 in Ruidera).

### 2.3 Diapausing egg isolation

A sediment subsample of 6 g (wet weight) from the center of each 2‐cm slice was taken for further processing. We used the modified sucrose flotation technique to isolate diapausing eggs from the sediments (Gómez et al., 2002). The supernatant was filtered through a 10 μm Nytal mesh and the filtrate washed with distilled water and transferred to a plankton counting chamber. We classified putative rotifer diapausing eggs according to their morphology (Koste, 1978) under an inverted microscope. Each egg was photographed prior to DNA analyses.

### 2.4 Identification of rotifers from water samples

Zooplankton samples were examined under an inverted microscope, and all individual rotifers were identified to species level using Koste's identification key (Koste, 1978).

### 2.5 DNA extraction, amplification, and sequencing

DNA was extracted from single adult rotifers or diapausing eggs using the hot sodium hydroxide and Tris (HotSHOT) method (Montero‐Pau, Gómez, & Muñoz, 2008). A region of the mitochondrial DNA from the cytochrome *c* oxidase subunit I gene (COI) was amplified using primers LCO1490 and HCO2198 (Folmer, Black, Hoch, Lutz, & Vrijenhoek, 1994). Each 50 μl PCR contained 5 μl of template DNA, 2 mM of MgCl_2_, 0.2 mM of each nucleotide, 0.2 μM of each primer, 5 μl of 10× NH_4_ Bioline buffer, and 0.5 U of *BioTaq* DNA polymerase (Bioline). Amplification was performed in a Veriti thermocycler (Applied Biosystems) with the following cycling profile: 1 cycle of 3 min at 93°C; 40 cycles of 15 s at 92°C, 20 s at 45°C, and 30 s at 70°C; and 3 min at 72°C. Samples giving clear strong single bands were sequenced in both directions by Macrogen Inc. (South Korea) using 3730XL DNA analyzer (Applied Biosystems, USA). Chromatograms were checked and edited using CodonCode Aligner (CodonCode Corporation, Dedham, MA).

### 2.6 Phylogenetic analysis

We constructed a first dataset that included all newly obtained sequences in this study (Dataset S1 hereafter). The aim of this dataset is, firstly, to identify diapausing eggs matching with their adults, which served as a reference collection of the study sites, as all individuals were identified to morphospecies; and secondly, to estimate the hidden biodiversity by detecting potential cryptic species. Sequences were aligned using the ClustalW algorithm included in ARB (Ludwig et al., 2004) and trimmed to 499 base pairs, the length of the shortest sequence in our alignment. The alignment was individually checked and verified for protein coding frameshifts to avoid pseudogenes using MEGA5 (Tamura et al., 2011). All sequences obtained in this study were submitted to GenBank with accession numbers (KY749339–KY749548).

To construct the second dataset (Dataset S2 hereafter), we downloaded all the available monogonont rotifer COI sequences from GenBank retrieved using the following search criteria: Monogononta organism, sequences over 300 bp with COI, CO1, COX, or cytochrome *c* oxidase subunit I in title (downloaded on 8 June 2016). The purpose of this dataset is to potentially match the cryptic species in our dataset to previously described ones available in the GenBank reference collection. We generated an alignment following the same procedure as described for Dataset S1. Identical sequences in both datasets were collapsed into haplotypes using the online fasta sequence toolbox FaBox v. 1.4 (Villesen, 2007) before phylogenetic analyses.

For both datasets, we reconstructed phylogenetic trees with maximum likelihood (ML) and Bayesian inference (BI). ML reconstructions were carried out using MEGA5 (Tamura et al., 2011). Nucleotide substitution model was chosen using the find best DNA/Protein models option in MEGA5 (Tamura et al., 2011). We used a sequence from the bdelloid rotifer *Philodina roseola* as an outgroup (GenBank accession number: DQ078544). BI reconstructions were performed in BEAST v. 1.8.3 (Drummond, Suchard, Xie, & Rambaut, 2012) as implemented on the CIPRES Science Gateway (Miller, Pfeiffer, & Schwartz, 2010). To do so, we used BEAUti v. 1.8.3 (Drummond et al., 2012) to create the xml input file needed for the BEAST runs with the following settings: a general time reversible with gamma distribution and invariable sites nucleotide substitution model (GTR + г + I) for Dataset S1 and a general time reversible with gamma distribution (GTR + г) for Dataset S2 with an uncorrelated lognormal relaxed clock. This model was implemented in BEAST, including an uncorrelated lognormal relaxed clock and coalescent prior, with the default settings of BEAUti for the remaining parameters. We created ultrametric phylogenies based on each COI dataset, and the phylogenetic analysis was run with two independent searches for 80,000,000 generations with trees sampled every 10,000 generations, with a total number of trees of 8,000 for Dataset S1. For Dataset S2, the phylogenetic analysis was run with two independent searches for 100,000,000 generations with trees sampled every 5,000. For examining that the effective sample size (ESS) values for all parameters were above 200 and determining the burn‐in, we used Tracer v. 1.6 in both datasets (Rambaut, Suchard, Xie, & Drummond, 2013). We obtained a total of 60,000 trees and summarized with TreeAnnotator v. 1.8.3; the first 50,000 trees were discarded as burn‐in.

### 2.7 DNA taxonomy

To identify the presence of independently evolving entities (putative cryptic species) in both COI datasets, we used the generalized mixed Yule coalescent method (GMYC), a robust tool for delimiting species using single‐locus data (Fontaneto et al., 2007; Fujisawa & Barraclough, 2013; Pons et al., 2006). It is a likelihood method designed to delimit independently evolving species by fitting within‐ and between‐species branching models to reconstructed gene trees. GMYC models were run on the previously constructed ultrametric maximum clade credibility consensus trees obtained with BEAST using R v. 3.3.1 (R Development Core Team, 2015) with the package *splits* v. 1.0‐11 (https://r-forge.r-project.org/projects/splits/).

We used a second, alternative approach of DNA taxonomy for delimiting species, the ABGD (http://wwwabi.snv.jussieu.fr/public/abgd/abgdweb.html). This method automatically identifies the threshold in genetic distances for species delimitation (a gap between intra‐ and interspecific diversity), the “barcoding gap” (Puillandre et al., 2012). We applied ABGD to Dataset S1 and Dataset S2 separately with all the sequences excluding the outgroup, because this method works better when there are more than 3–5 sequences per species. We used the uncorrected, JC69 and K2P distance matrices with default options (Pmin: 0.001, Pmax: 0.1, steps: 10, Nb bins: 20), except for the relative gap width (*X*) that was set to 1. Higher values than 1 recovered only one cluster. We considered only the results with a prior intraspecific divergence higher than 1.5%, as it has previously been described in rotifers for COI (Fontaneto, 2014).

DnaSP v4.0 (Rozas & Rozas, 1995) was used to calculate DNA divergence between the lineages delimited by both GMYC and ABGD analyses in Dataset S1 for groups of putative cryptic species.

## 3 Results

### 3.1 Sample processing and molecular analyses

**Figure 2:**
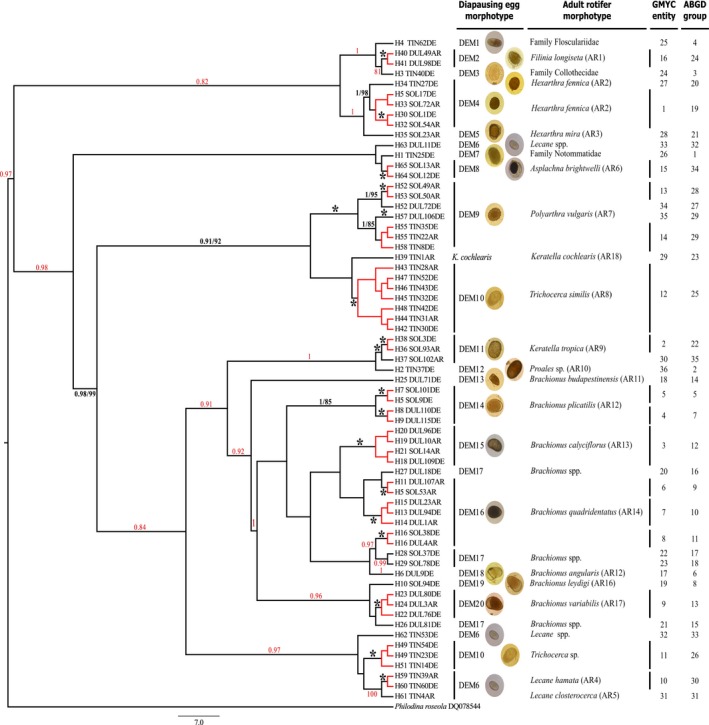
Phylogenetic relationships of the 65 COI rotifer haplotypes newly obtained, according to Bayesian Inference reconstructions. The consensus of 10,000 sampled trees from BI constructed with BEAST using the general time reversible  + G + I model. Branch length indicates number or substitutions per site. Posterior probabilities values from the BI reconstruction above 0.8 and 80 for bootstrap support from the ML reconstruction are shown in each branch, respectively. (*) Asterisks indicate values of posterior probability and bootstrap support of 1 and 100 respectively. Values in red show posterior probability or bootstrap support above 0.8 and 80 respectively. Black branches indicate delineated OTUs, and red lines represent haplotypes belonging to the same GMYC entity (OTUs). Diapausing egg morphotype (DEM); adult rotifer morphotype (AR). The number of potential OTUs within each species according to the different methods in DNA taxonomy (ABGD and GMYC on different chronograms) is reported. Monophyletic groups in red indicate a single putative species recognized by the GMYC analysis. Note that the actual samples for each haplotype are detailed in Appendix S1

The processing of 12 g (wet weight) of sediment in each lake resulted in a total of 193 putative rotifer diapausing eggs (Santa Olalla 6.08 eggs/g, Dulce 6.58 eggs/g, and Tinaja 3.41 eggs/g). These eggs were classified into 20 different groups according to their morphology (Table 1 and Figure 2). DNA was successfully extracted and COI amplified from 151 eggs (78.2% success rate), a high percentage given that it is difficult to identify healthy diapausing eggs in some species (García‐Roger, Carmona, & Serra, 2006). Additionally, we identified 18 taxonomic species from the water‐column samples according to their morphology. Only 59 of 289 zooplankton samples yielded successful amplifications (20.4%), probably due to poor preservation (as opposed to poor primer match), as we managed to obtain high quality sequences from at least one specimen of all 18 taxonomic species identified. These samples were then used as a reference collection to assign diapausing eggs to particular taxonomic species by DNA barcoding.

**Table 1:** Correspondence between diapausing egg morphotypes (DEM) and taxonomic species after DNA taxonomy

Diapausing egg morphotypes	*N* seq	*N* DE	*N* AR	*H*	GMYC entities	Taxon	Location
DEM1	1	1	0	1	1	Fam. Flosculariidae	Tinaja
DEM2	15	12	3	2	1	*Filinia longiseta*	Santa Olalla, Dulce
DEM3	1	1	0	1	1	Fam. Collothecidae	Tinaja
DEM4	9	6	3	5	2	*Hexarthra fennica*	Santa Olalla, Tinaja
DEM5	7	5	2	1	1	*Hexarthra mira*	Dulce
DEM6	9	7	2	4	4	*Lecane* spp.	Dulce, Tinaja
DEM7	1	1	0	1	1	Fam. Notommatidae	Tinaja
DEM8	10	2	8	2	1	*Asplanchna brightwellii*	Santa Olalla
DEM9	17	13	4	7	4	*Polyarthra vulgaris*	Santa Olalla, Dulce, Tinaja
DEM10	15	13	2	4	2	*Trichocerca* spp.	Tinaja
DEM11	17	12	5	3	2	*Keratella tropica*	Santa Olalla, Dulce
DEM12	2	1	1	1	1	*Proales* sp.	Tinaja
DEM13	2	1	1	2	1	*Brachionus budapestinensis*	Dulce
DEM14	46	28	18	3	2	*Brachionus plicatilis*	Santa Olalla, Dulce
DEM15	17	11	6	5	1	*Brachionus calyciflorus*	Santa Olalla, Dulce
DEM16	12	5	7	8	3	*Brachionus quadridentatus*	Santa Olalla, Dulce, Tinaja
DEM17	4	4	0	3	4	*Brachionus* spp.	Santa Olalla, Dulce
DEM18	10	7	3	1	1	*Brachionus angularis*	Santa Olalla, Dulce
DEM19	4	3	1	1	1	*Brachionus leydigi*	Santa Olalla
DEM20	7	6	1	3	1	*Brachionus variabilis*	Santa Olalla, Dulce

Total number of sequences obtained for each monogonont rotifer diapausing egg morphotype and morphospecies (*N* seq), number of sequences of each diapausing egg (*N* DE), number of sequences of each monogonont adult rotifer morphospecies (*N* AR), number of haplotypes (*H*), GMYC entities, and lakes from which samples were collected at each location for COI rotifer sequences (Location).

### 3.2 Estimation of rotifer taxonomic diversity from the diapausing egg bank

We obtained a total of 210 new sequences from all three locations from both adults and diapausing eggs (listed in Appendix S1). Sequences contained neither stop codons nor indels and were aligned unambiguously and collapsed into 65 haplotypes. A first approach for adult and diapausing egg morphotype assignation was based on ML and BI phylogenetic analyses (Figure 2). The consensus of 10,000 sampled trees from BI constructed with BEAST using the general time reversible + G + I model is shown in Figure 2. The GMYC model suggested the presence of 36 entities in Dataset S1 (clusters plus singletons and excluding outgroups with a confidence interval 34–38) and was the most likely model (likelihood of null model: 251.9922, ML of GMYC model: 293.8043, likelihood ratio: 83.62426, LR test: <0.0001). Sequences from diapausing eggs belonged to 35 GMYC entities, while zooplankton sample belonged to 24 entities (see Table 1 and Figure 2). These results showed how each diapausing egg morphotype belonged to a different GMYC entity, but also that diapausing egg morphotypes can include one or more GMYC entities. Diapausing eggs were assigned to a taxonomical species when haplotypes from both the egg and the identified adult belonged to the same GMYC entity. In this way, eleven of twelve Santa Olalla diapausing egg morphotypes belonged to the same operational taxonomic unit (OTU) as an adult in the dataset, and only a single diapausing egg belonging to the *Brachionus* sp. morphotype was unidentified. In Dulce, nine of thirteen diapausing egg morphotypes were assigned to the same OTU as rotifer adult morphospecies. The remaining three, which had no assignment with adults, were classified into genera *Brachionus*,* Lecane*, and *Polyarthra*. In Tinaja, six of nine diapausing egg morphotypes were identified to species level with their adult rotifer isolated from the water samples. Sequences from diapausing egg morphotype *Brachionus quadridentatus* were grouped within a group of sequences from Dulce and Santa Olalla, and the *Proales* sp. diapausing egg morphotype were classified to genus level. In summary, 85% of sequences from diapausing eggs were assigned to a taxonomic species (17 of the 20 diapausing eggs morphotypes, Santa Olalla 91%, Dulce 69%, and Tinaja 66%; we excluded the adult rotifer *K. cochlearis*).

The most widely represented genus in our dataset was *Brachionus* with 14 GMYC entities, 8 *Brachionus* species in Santa Olalla and Dulce lakes: Two are *B. plicatilis* which are followed in order of abundance by *B. calyciflorus*,* Brachionus angularis*,* Brachionus variabilis*,* Brachionus budapestinensis*,* Brachionus leydigi*, and *B. quadridentatus*, the latter containing three independent GMYC entities, one of them containing sequences from Doñana and Ruidera. The genus *Hexarthra* showed two main species, *Hexarthra mira* and *Hexarthra fennica* in Doñana, and a third *H. fennica* from Tinaja sediment samples, which corresponded to a different GMYC entity. From the genus *Polyarthra*,* Polyarthra vulgaris* was made of three GMYC entities from all study site samples and an unidentified *Polyarthra* single diapausing egg from Dulce. The common genus *Lecane* showed four GMYC entities, two of them from Tinaja Lake included *Lecane closterocerca* and *Lecane hamata* adult rotifers. The other two *Lecane* GMYC entities corresponded to the same diapausing egg morphotype and one included a single diapausing egg from Dulce Lake, and the other contained two diapausing eggs from Tinaja and Dulce lakes. *Keratella tropica* from both Doñana lakes split in two GMYC entities. A group of diapausing eggs identified as *Trichocerca* type formed an independent GMYC entity from *Trichocerca similis*. Seven diapausing egg sequences (three from Santa Olalla and two from Dulce with *Brachionus* diapausing egg morphotype, and three unidentified diapausing egg morphotypes from Tinaja) showed seven GMYC entities with no adult rotifer assignation. Additional species that did not split into different GMYC entities in our dataset were *Asplanchna brightwellii*,* Filinia longiseta*, and *Proales* sp. Similarly, sequences from adult *K. cochlearis* from Tinaja Lake lacked corresponding diapausing egg sequences. Uncorrected *p*‐distances between each pair of GMYC entities considered as potential cryptic species varied from the lowest value .0378 of *K. tropica* to the highest .2431 of *H. fennica* (see Table S1).

The ABGD analysis indicated a clear barcode gap between 0.05 and 0.12 for Dataset S1 and grouped the sequences into 35 groups (see Table S2 and Figure S1), 34 if we only considered the diapausing eggs. The choice between alignments or distance matrix (uncorrected, JC or K2P) had little influence on the results (see Table S2). The groupings delimited by ABGD were highly congruent with the most conservative result of the GMYC analyses (Table 2).

**Table 2:** Number of GMYC entities includes the most likely solution and confidence interval of Dataset S1 and Dataset S2

Dataset	Clusters	GMYC entities	Likelihood‐null	Likelihood‐GMYC	Likelihood ratio	Threshold	GMYC entities (conservative results)	ABGD groups
1	16	36 (34–38)	251.9922	293.8043	83.62426	−0.020325	36	35
2	148	285 (271–306)	11089.35	11239.9	301.1003	−0.035099	271/37^a^	252/37^a^

a Number of GMYC entities and ABGD groups obtained from our sequences in Dataset S2 analyses.

### 3.3 Cryptic species

For the second dataset, we downloaded the available 3,597 monogonont rotifer COI sequences from GenBank following the criteria described above. To this we added the 210 sequences obtained in this study both from diapausing eggs and adult rotifers. There were a total of 499 positions in the final alignment, after trimming and removing 120 shorter sequences. Sequence alignments and amino acid translations were unambiguous, with no gaps or stop codons among the 3,686 sequences. A total of 427 polymorphic sites, including 352 parsimony informative sites, defined 1,275 unique haplotypes. The GMYC analysis for Dataset S2, resulted in 148 GMYC entities with strong support, and a total number of 285 GMYC entities (clusters plus singletons and excluding outgroups with a confidence interval 271–306) and it was the most likely model (likelihood of null model: 11089.35, ML of GMYC model: 11239.9, likelihood ratio: 301.1003, LR test: <0.0001). (see Figure S3 and Appendix S2, supporting information) ABGD analyses generated 253 groups in the recursive partitions (see Table S2 and Figure S2). The two *B. plicatilis* GMYC entities found in both Doñana lakes were identified as “Almenara” and “Tiscar” belonging to the *B. plicatilis* complex. The GMYC entities with no adult rotifer assignation, TIN25DE, TIN40DE, and TIN62DE (DE, diapausing egg sequence), were identified to Notonmatidae, Collothecidae, and Flosculariidae family (*Floscularia melicerta*). The GMYC entity corresponding to the diapausing eggs DUL24DE and TIN53DE from Dulce and Tinaja respectively and the GMYC entity, which included the single DUL11DE sequence, belonged to the group of *Lecane* spp. species. The *Lecane* sp. diapausing egg morphotype identified as *L. closterocerca* and *L. hamata* was closely related with other *L. closterocerca* and *L. hamata*, supporting the idea that *Lecane* group is a huge species complex. *Polyarthra vulgaris* from Tinaja and Dulce were included in the same GMYC entity with an unpublished sequence of *Polyarthra* sp. The other *P. vulgaris* GMYC entity were closely related to the last GMYC entity and to the only *Polyarthra* diapausing egg morphotype from Dulce Lake. All three GMYC entities seem to be related to the *P. dolichoptera* species complex (Obertegger et al., 2014). *Keratella cochlearis* from Tinaja Lake belonged to the same GMYC entity that an unpublished *K. cochlearis* sequence. Our *B. calyciflorus* sequences were included in the same GMYC entity than *B. calyciflorus* sequences from the Netherlands and China. *Brachionus quadridentatus* showed three GMYC entities, one including a *B. quadridentatus* f. *cluniorbicularis* from Mexico. The four *Brachionus* diapausing egg morphotypes were confirmed.

We compared the number of taxa estimated from the diapausing egg banks and plankton sample with zooplankton species lists which included rotifer species compiled in previous studies from the three locations (Table 3). Our approach retrieved higher number of taxa for both Doñana lakes than in previous studies using conventional sampling techniques in the zooplankton (Galindo, Mazuelos, Mata, & Serrano, 1994; Guisande, Granado‐Lorencio, Toja‐Santillana, León, & León‐Muez, 2008; López et al., 1991). However, in Tinaja Lake, our approaches estimated fewer taxa both in the egg bank and in the water column than classical methods in a long term sampling campaign which included one sample every 2–3 months over 3 years (Moreno et al., in prep) and other studies (Álvarez‐Cobelas et al., 2007).

**Table 3:** Total number of GMYC entities, GMYC entities retrieved from diapausing egg banks and from zooplankton samples

Location	GMYC entities			ABGD			Plankton samples (other works)
	Total entities	Sediment sample	Plankton sample	Total groups	Sediment sample	Plankton sample	
Tinaja	14	12	7	14	12	7	18 morphospecies and 15 samples (2008–2011)^a^25 morphospecies and four samples (2001–2002)^b^
Santa Olalla	18	14	14	18	14	14	12 morphospecies and 17 samples (2008–2011)^a^ 7 morphospecies and 21 samples (1989–1992)^c^ 4 morphospecies and 23 samples (1985–1987)^d^ 5 morphospecies and six samples (2004)^e^
Dulce	19	18	12	19	18	12	16 morphospecies and 17 samples (2008–2011)^a^ 11 morphospecies and 21 samples (1989–1992)^c^ 11 morphospecies and 23 samples (1985–1987)^d^ 8 morphospecies and six samples (2004)^e^

Number of taxonomic species and samples from previous work in our study sites.

a Moreno et al. in prep.

b Álvarez‐Cobelas et al., 2007

c Galindo et al., 1994

d López et al., 1991

e Guisande et al., 2008

## 4 Discussion

This is the first study using DNA barcoding on diapausing egg banks from a single sediment sample to characterize rotifer communities in continental aquatic systems. We generated a reference collection by barcoding individuals sampled from the water column, to which we assigned sequences obtained from diapausing eggs from sediments. Our DNA taxonomy analysis yielded 35 GMYC entities in the diapausing egg bank, a substantially higher number than the 20 types of diapausing eggs previously identified based on morphology. We identified 61% of GMYC entities to taxonomic species level and a 91% to likely genus level, with a combination of two reference data sets, one generated in this study and one downloaded from GenBank. Our approach, based on a single sediment sample, gave higher or similar estimates of rotifer biodiversity than previous studies based on a number of seasonal samples, reducing time and sampling effort.

### 4.1 Temporary versus permanent environments

The success of the application of barcoding to diapausing egg banks might differ between ponds with different hydrology, as this is known to influence the investment of rotifer species into diapausing eggs. Seasonal environments with fluctuating conditions may induce a higher diapausing egg production (Altermatt & Ebert, 2008; Cáceres & Tessier, 2004; García‐Roger, Carmona, & Serra, 2005; Pérez‐Martínez, Barea‐Arco, Conde‐Porcuna, & Morales‐Baquero, 2007). Indeed, Santa Olalla and Dulce show drastic hydrological fluctuations between years (Moreno et al., 2016) and both Doñana Lakes present a higher abundance of diapausing eggs in comparison with Ruidera. In contrast, in permanent habitats dormancy might not be strictly necessary, and asexual reproduction would be favored (Serra & King, 1999; Schröder, 2005); therefore, some common rotifer species in more permanent environments may either lack a diapausing egg stage or produce few diapausing eggs (for instance, as we observed for *K. cochlearis* from Tinaja Lake, and previously reported by Dumont, 1983). In consequence, DNA barcoding would be expected to be a more useful tool on diapausing egg banks in temporal–semipermanent systems than in permanent ones. Therefore, in the case of permanent and stable water bodies such as Ruidera Lakes, this molecular technique should be performed using both sediment and water samples simultaneously to include diapausing eggs and adults and obtain a good characterization of the rotifer community.

### 4.2 Reference collections

To aid in the identification of the diapausing eggs obtained, it is necessary to compare the sequences obtained to a curated reference collection of barcodes. For this, we (i) gathered a representative and taxonomically identified rotifer species list from the water column of the sampled lakes as a reference collection for rotifer diapausing egg bank characterization of the local area and (ii) we compared our dataset with rotifers sequences published in GenBank to identify cryptic species previously described. We avoided the limitations of using hatching from sediment samples, as optimal hatching cues can be species‐specific and bet‐hedging might limit the number of viable eggs that hatch from a given sample (García‐Roger et al., 2014; Schröder, 2005; Vandekerkhove, Declerck, Brendonck, Conde‐Porcuna, Jeppesen and De Meester, 2005), as well as the undescribed diversity of diapausing egg morphologies (Gilbert & Wurdak, 1978; Snell, Burke, & Messur, 1983; Munuswamy et al. 1996). Therefore, DNA barcoding might solve a misdiagnosis but we need a reference collection of barcodes.

Using diapausing eggs as proxies to estimate biodiversity (Pourriot & Snell, 1983; Ricci, 2001) might incur biases due to the lack of diapausing egg production in some species or habitat heterogeneity. Hence, some discrepancies between sediment samples and plankton samples are expected, they reflect the differences in sampling intensity (zooplankton survey vs sediment sample). A single sediment sample might reflect the biodiversity or composition of a diapausing egg bank, but cannot reflect their habitat heterogeneity (Chittapun, Pholpunthin, & Segers, 2005). In addition, the usefulness of the combination of collecting plankton and sediment samples can be improved using DNA barcoding (Hebert et al., 2003).

### 4.3 Cryptic species

The species list made for our study sites reveals the presence of three rotifer taxonomic species that are part of well studied and described cryptic complexes (*B. plicatilis*,* B. calyciflorus* and *P. vulgaris*) and four potential cryptic complexes (*B. quadridentatus*,* K. tropica*,* H. fennica* and *Lecane* spp.). To verify the presence of cryptic species we used DNA taxonomy analyses, which provided higher taxonomic resolution than morphological identification. GMYC identified 36 entities from 17 adults and 20 different morphological diapausing eggs. ABGD also clustered the sequences into 35 groups congruent with the most conservative GMYC result. *Brachionus plicatilis* from Doñana samples and *B. quadridentatus* from Doñana and Ruidera samples belonged to different OTUs. The rest of *Brachionus* species with both diapausing egg and adult samples were *B. angularis*,* B. budapestinensis*,* B. leydigi*, and *B. variabilis*. The remaining four *Brachionus* groups were singletons from diapausing eggs without adult confirmation. Those results reveal the widespread presence of this genus in these freshwater systems and its importance in zooplankton communities. In the *Keratella* group, *K. tropica* splits in two groups with strong support, suggesting the presence of potential cryptic species in both Doñana lakes. Other diverse group was the genus *Lecane*, which seems to comprise numerous undescribed cryptic species complexes (García‐ Morales & Elías‐Gutiérrez, 2013; Walsh et al., 2009). The absence of Colothecaceae and Flosculariidae adults in the water‐column samples is not surprising as these are may be related with the species of the family Collothecidae, which are mainly benthonic (De Manuel, 2000). The absence of corresponding sequences from adults from the water column for some of the GMYC entities found could also be due to the fact that a single plankton samples was used to build out reference collection.

The application of GMYC and ABGD analysis to Dataset S2 which included all the monogonont rotifer COI sequences available in GenBank in addition to our sequences, revealed that some cryptic species in our dataset belonged to previously described species complexes (García‐Morales & Elías‐Gutiérrez, 2013; Gómez et al., 2002; Obertegger et al., 2014; Xiang et al., 2011). The most studied rotifer cryptic species complexes, *B. plicatilis* and *B*. *calyciflorus*, split in 33 and 12 entities, respectively in Dataset S2. The *B. calyciflorus* entity found in Doñana samples belonged to the group of BcwII published by Xiang et al. (2011), probably a cosmopolitan species also collected in the Netherlands and China. In Doñana, Dulce, and Santa Olalla lakes, the most abundant monogonont rotifer was *B. plicatilis* in both sediment and freshwater samples, which split in “Almenara” and “Tiscar” species (Gómez et al., 2002; Ortells, Gómez, & Serra, 2006). *Brachionus* “Almenara” and *Brachionus* “Tiscar” were previously found by Ortells et al. (2006) in Mediterranean ponds, together with *B. plicatilis* “sensu stricto” also found in Doñana brackish water ponds (Badosa, Frisch, Green, Rico, & Gómez, 2017). Other *Brachionus* species which seems to form a species complex is *B. quadridentatus*, which includes three independent OTUs, two from Doñana lakes and a third one from Doñana and Ruidera. One of these OTUs includes a *B. quadridentatus* sequence from Mexico (García‐Morales & Elías‐Gutiérrez, 2013). In addition, sequences belonging to the morphospecies *P. vulgaris* from Doñana and Ruidera split into four OTUs. We also found for the first time a potential cryptic group of *Hexarthra* species, since that *H. fennica* from Doñana and Ruidera split in two OTUs and comprises a monophyletic group with strong support. The sequences we obtained for *Hexarthra* species are the first ones available (GenBank accession numbers X–X). All individuals of *H. fennica* from diapausing eggs and adult samples were morphologically homogeneous; nevertheless, we identified two potentially cryptic species based on GMYC and ABGD analyses, with genetic distances of 0.2431, one located in Tinaja Lake from Ruidera, and the other located in Santa Olalla Lake from Doñana.

Our study underscores the common coexistence of zooplanktonic cryptic species in rotifers (Gabaldón et al., 2016; Gómez, Montero‐Pau, Lunt, Serra, & Campillo, 2007; Gómez et al., 2002; Li, Niu, & Ma, 2010; Obertegger et al., 2012; Ortells, Gómez, & Serra, 2003). *Brachionus plicatilis* species complex is the best studied group of cryptic species with a well described representation of their coexistence in the Iberian Peninsula (Gómez et al., 2002). We found *Brachionus* “Tiscar” and *Brachionus* “Almenara” coexisting in two nearby lakes (Santa Olalla and Dulce). These species have been previously described in Mediterranean lakes, but they have never been found coexisting in the same lake (Gómez et al., 2002; Montero‐Pau, Ramos‐Rodríguez, Serra, & Gómez, 2011; Ortells et al., 2006). “Almenara” was restricted to coastal lagoons of low salinity and “Tiscar” has been found in inland and coastal lakes. There is no evidence of both species co‐occurring in the same water body. We show for the first time their coexistence of these cryptic species either in water column and sediments in Santa Olalla and Dulce lakes, although it is not clear if both cryptic species coexist at the same time in the water column.

An emerging and highly promising molecular approach and alternative technique to DNA barcoding zooplankton communities might be DNA metabarcoding (e.g., see Deiner, Fronhofer, Mächler, Walser, & Altermatt, 2016). DNA metabarcoding is a high‐throughput multispecies‐identification tool from a single bulk sample containing entire organisms or degraded DNA (Taberlet, Coissac, Pompanon, Brochmann, & Willerslev, 2012). However, the application of DNA metabarcoding to rotifer diapausing eggs has some constraints. First, the multispecies‐identification approach requires that the primers used are highly versatile to avoid biases in species amplification, but at the same time specific to the group of interest. Second, quantitative information is difficult to extract from the sequence information. Third, no morphological information can be linked to each individual. Despite these problems, the increasingly used and cost‐effective of DNA metabarcoding might eventually become an effective tool applied to zooplankton diapausing egg banks characterization.

## 5 Conclusion

Our results highlight how an integrated taxonomic approach, combining DNA barcoding of diapausing eggs from a single sediment sample with the rapidly improving rotifer reference collection of DNA barcodes can be an efficient method to characterize rotifer communities from lentic aquatic systems. These results agree with previous studies that have shown how the zooplankton community characterization from diapausing egg banks is more effective than intensive samplings of active communities from different seasons (Duggan et al., 2002; May, 1986; Vandekerkhove et al., 2004), reducing time and sampling effort. Our molecular approach, based on DNA barcoding and DNA taxonomy, solve the problems related to diapausing eggs morphological identification and hatching cues and not only correctly classified a high percentage of the rotifer diapausing egg sequences, but also revealed the occurrence of cryptic species overlooked by traditional taxonomic methods. The incorporation of GMYC analysis for delimiting species boundaries as a complementary tool for DNA barcoding facilitated the identification of new cryptic species. Although GMYC has the tendency to oversplit in comparison with ABGD (Tang et al., 2012), both techniques gave similar results, resulting in accurate species delimitation and the presence of potentially undescribed cryptic species. DNA taxonomy for species delimitation should be considered as a first step for taxonomic identifications instead of a conclusive taxonomic tool (Kekkonen & Hebert, 2014). Nevertheless, we support the idea of integrative taxonomic approach as DNA barcoding might be a powerful complementary tool, in such cases where rotifer species do not produce diapausing eggs or they are produced in very low quantities and problems with the DNA preservation or amplification. Finally, our understanding of rotifer communities from diapausing egg banks would benefit tremendously from the further development of the rotifer reference database of barcodes.

## Author Contributions

E. Moreno sampled the zooplankton samples and collected the sediment cores, analyzed the data, and wrote the manuscript. J.M. Conde‐Porcuna supported the fieldwork and wrote the manuscript. A. Gómez designed and performed the research, analyzed the data, and wrote the manuscript.

## Data Accessibility

DNA sequences: GenBank accessions: KY749339–KY749548. Final DNA sequence assembly of Dataset S1 and Dataset S2: online Supporting Information.

## Conflicts of Interest

Nothing to declare.

## References

[ece32986-bib-0001] Altermatt, F. , & Ebert, D. (2008). The influence of pool volume and summer desiccation on the production of the resting and dispersal stage in a *Daphnia* metapopulation. Oecologia, 157, 441–452. 18597121 10.1007/s00442-008-1080-4

[ece32986-bib-0002] Álvarez‐Cobelas, M. , Cirujano, S. , Montero, E. , Rojo, C. , Rodrigo, M. A. , Piña, E. , … Araujo, R. (2007) Aquatic ecology and society of Ruidera lakes (Central Spain). Madrid: Publicaciones CSIC, 414.

[ece32986-bib-0003] Badosa, A. , Frisch, D. , Green, A. J. , Rico, C. , & Gómez, A. (2017). Isolation mediates persistent founder effects on zooplankton colonisation in new temporary ponds. Scientific Reports, 7, https://doi.org/10.1038/srep43983. 10.1038/srep43983 PMC5343421 28276459

[ece32986-bib-0004] Birky, C. W. , Wolf, C. , Maughan, H. , Herbertson, L. , & Henry, E. (2005). Speciation and selection without sex. Hydrobiologia, 546, 29–45.

[ece32986-bib-0005] Bort, S. , Rojo, C. , Rodrigo, M. A. , & Maidana, N. (2005). El fitoplancton de las lagunas de Ruidera (Parque Natural, Ciudad Real). Limnetica, 24, 33–46.

[ece32986-bib-0006] Brendonck, L. , & De Meester, L. (2003). Egg banks in freshwater zooplankton: Evolutionary and ecological archives in the sediment. Hydrobiologia, 491, 65–84.

[ece32986-bib-0007] Briski, E. , Cristescu, M. E. , Bailey, S. A. , & MacIsaac, H. J. (2011). Use of DNA barcoding to detect invertebrate invasive species from diapausing eggs. Biological Invasions, 13, 1325–1340.

[ece32986-bib-0008] Cáceres, C. E. , & Tessier, A. J. (2004). To sink or swim: Variable diapause strategies among *Daphnia* species. Limnology and Oceanography, 9, 1333–1340.

[ece32986-bib-0009] Chittapun, S. , Pholpunthin, P. , & Segers, H. (2005). Restoration of tropical peat swamp rotifer communities after perturbation: An experimental study of recovery from resting egg bank. Hydrobiologia, 546, 281–289.

[ece32986-bib-0010] Cieplinski, A. , Weisee, T. , & Obertegger, U. (2016). High diversity in *Keratella cochlearis* (Rotifera, Monogononta): Morphological and genetic evidence. Hydrobiologia, 1–15. https://doi.org/10.1007/s10750-016-2781-z.

[ece32986-bib-0011] De Manuel, J. (2000). The rotifers of Spanish reservoirs: Ecological, systematical and zoogeographical remarks. Limnetica, 19, 91–167.

[ece32986-bib-0012] Deiner, K. , Fronhofer, E. A. , Mächler, E. , Walser, J.‐C. , & Altermatt, F. (2016). Environmental DNA reveals that rivers are conveyer belts of biodiversity information. Nature Communications, 7, 12544. 10.1038/ncomms12544 PMC5013555 27572523

[ece32986-bib-0013] Derry, A. , Hebert, P. D. N. , & Prepas, E. E. (2003). Evolution in saline and subsaline lakes: A molecular phylogenetic approach. Limnology and Oceanography, 48, 675–685.

[ece32986-bib-0014] DeStasio, B. T. (1989). The seed bank of a freshwater crustacean: Copepodology for the plant ecologist. Ecology, 70, 1377–1389.

[ece32986-bib-0015] Drummond, A. J. , Suchard, M. A. , Xie, D. , & Rambaut, A. (2012). Bayesian phylogenetics with BEAUti and the BEAST 1.7. Molecular Biology and Evolution, 29, 1969–1973. 22367748 10.1093/molbev/mss075 PMC3408070

[ece32986-bib-0016] Dudgeon, D. , Arthington, A. H. , Gessner, M. O. , Kawabata, Z.‐I. , Knowler, D. J. , Lévêque, C. , … Sullivan, C. A. (2006). Freshwater biodiversity: Importance, threats, status and conservation challenges. Biological Review, 81, 163–182. 10.1017/S1464793105006950 16336747

[ece32986-bib-0017] Duggan, I. C. , Green, J. D. , & Shiel, R. J. (2002). Rotifer egg densities in lakes of different trophic state, and their assessment using emergence and egg counts. Archiv für Hydrobiologie, 153, 409–420.

[ece32986-bib-0018] Dumont, H. J. (1983). Biography of rotifers. Hydrobiologia, 104, 19–30.

[ece32986-bib-0019] Esteban, G. F. , & Finlay, B. J. (2010). Conservation work is incomplete without cryptic biodiversity. Nature, 463, 293. 10.1038/463293c 20090730

[ece32986-bib-0020] Fahd, K. , Florencio, M. , Keller, C. , & Serrano, L. (2007). The effect of the sampling scale on zooplankton community assessment and its implications for the conservation of temporary ponds in southwest Spain. Aquatic Conservation: Marine and Freshwater Ecosystems, 17, 175–193.

[ece32986-bib-0021] Folmer, O. , Black, M. , Hoch, W. , Lutz, R. , & Vrijenhoek, R. (1994). DNA primers for amplification of mitochondrial cytochrome *c* oxidase subunit 1 from diverse metazoan invertebrates. Molecular Marine Biology and Biotechnology, 3, 294–299. 7881515

[ece32986-bib-0022] Fontaneto, D. (2014). Molecular phylogenies as a tool to understand diversity in rotifers. International Review of Hydrobiology, 99, 178–187.

[ece32986-bib-0023] Fontaneto, D. , Barraclough, T. G. , Chen, K. , Ricci, C. , & Herniou, E. A. (2008). Molecular evidence for broad‐scale distributions in bdelloid rotifers: Everything is not everywhere but most things are very widespread. Molecular Ecology, 17, 3136–3146. 18522694 10.1111/j.1365-294X.2008.03806.x

[ece32986-bib-0024] Fontaneto, D. , Flot, J.‐F. , & Tang, C. Q. (2015). Guidelines for DNA taxonomy with a focus on the meiofauna. Marine Biodiversity, 45, 433–451.

[ece32986-bib-0025] Fontaneto, D. , Herniou, E. A. , Boschetti, C. , Caprioli, M. , Melone, G. , Ricci, C. , & Barraclough, T. G. (2007). Evidence for independently evolving species in bdelloid rotifers. PLoS Biology, 5, 914–921. 10.1371/journal.pbio.0050087 PMC1828144 17373857

[ece32986-bib-0026] Fontaneto, D. , Iakovenko, N. , Eyres, I. , Kaya, M. , Wyman, M. , & Barraclough, T. G. (2011). Cryptic diversity in the genus *Adineta* Hudson & Gosse, 1886 (Rotifera: Bdelloidea: Adinetidae): A DNA taxonomy approach. Hydrobiologia, 662, 27–33.

[ece32986-bib-0027] Fontaneto, D. , Kaya, M. , Herniou, E. A. , & Barraclough, T. G. (2009). Extreme levels of hidden diversity in microscopic animals (Rotifera) revealed by DNA taxonomy. Molecular Phylogenetics and Evolution, 53, 182–189. 19398026 10.1016/j.ympev.2009.04.011

[ece32986-bib-0028] Fujisawa, T. , & Barraclough, T. G. (2013). Delimiting species using single‐locus data and the generalized mixed Yule coalescent (GMYC) approach: A revised method and evaluation on simulated datasets. Systematic Biology, 29, 2869–2876. 10.1093/sysbio/syt033 PMC3739884 23681854

[ece32986-bib-0029] Gabaldón, G. , Fontaneto, D. , Carmona, M. J. , Montero‐Pau, J. , & Serra, M. (2016). Ecological differentiation in cryptic rotifer species: What we can learn from the *Brachionus plicatilis* complex. Hydrobiologia, 1–12. https://doi.org/10.1007/s10750-016-2723-9

[ece32986-bib-0030] Gabaldón, C. , Serra, M. , Carmona, M. J. , & Montero‐Pau, J. (2015). Life‐history traits, abiotic environment and coexistence: The case of two cryptic rotifer species. Journal of Experimental Marine Biology and Ecology, 465, 142–152.

[ece32986-bib-0031] Galindo, M. D. , Mazuelos, N. , Mata, A. J. , & Serrano, L. (1994). Microcrustacean and rotifer diversity relating to water temporality in dune ponds of the Doñana National Park. Verhandlungen der Internationalen Vereinigung für Theoretische und Angewandte Limnologie, 25, 1350–1356.

[ece32986-bib-0032] García‐Morales, A. E. , & Elías‐Gutiérrez, M. (2013). DNA barcoding of freshwater Rotifera in Mexico: Evidence of cryptic speciation in common rotifers. Molecular Ecology Resources, 13, 1097–1107. 23433240 10.1111/1755-0998.12080

[ece32986-bib-0033] García‐Roger, E. M. , Carmona, M. J. , & Serra, M. (2005). Deterioration patterns in diapausing egg banks of *Brachionus* (Muller, 1786) rotifer species. Journal of Experimental Marine Biology and Ecology, 314, 149–161.

[ece32986-bib-0034] García‐Roger, E. M. , Carmona, M. J. , & Serra, M. (2006). Hatching and viability of rotifer diapausing eggs collected from pond sediments. Freshwater Biology, 51, 1351–1358.

[ece32986-bib-0035] García‐Roger, E. M. , Serra, M. , & Carmona, M. J. (2014). Bet‐hedging in diapausing egg hatching of temporary rotifer populations – a review of models and new insights. International Review of Hydrobiology, 99, 96–106.

[ece32986-bib-0036] Gilbert, J. J. (1974). Dormancy in rotifers. Transactions of the American Microscopical Society, 93, 490–512.

[ece32986-bib-0037] Gilbert, J. J. (1995). Structure, development and induction of a new diapause stage in rotifers. Freshwater Biology, 34, 263–270.

[ece32986-bib-0038] Gilbert, J. J. , & Walsh, E. J. (2005). *Brachionus calyciflorus* is a species complex: Mating behavior and genetic differentiation among four geographically isolated strains. Hydrobiologia, 546, 257–265.

[ece32986-bib-0039] Gilbert, J. J. , & Wurdak, E. S. (1978). Species‐specific morphology of resting eggs in the rotifer *Asplanchna* . Transactions of the American Microscopical Society, 97, 330–339. 564567

[ece32986-bib-0040] Gillooly, J. F. (2000). Effect of body size and temperature on generation time in zooplankton. Journal of Plankton Research, 22, 241–251.

[ece32986-bib-0041] Gleason, R. A. , Euliss, N. H. , Hubbard, D. E. , & Duffy, W. G. (2004). Invertebrate egg banks of restored, natural, and drained wetlands in the prairie pothole region of the United States. Wetlands, 24, 562–572.

[ece32986-bib-0042] Gómez, A. , Montero‐Pau, J. , Lunt, D. H. , Serra, M. , & Campillo, S. (2007). Persistent genetic signatures of colonization in *Brachionus manjavacas* rotifers in the Iberian Peninsula. Molecular Ecology, 16, 3228–3240. 17651199 10.1111/j.1365-294X.2007.03372.x

[ece32986-bib-0043] Gómez, A. , Serra, M. , Carvalho, G. R. , & Lunt, D. H. (2002). Speciation in ancient cryptic species complex: Evidence from the molecular phylogeny of *Brachionus plicatilis* (Rotifera). Evolution, 56, 1431–1444. 12206243 10.1111/j.0014-3820.2002.tb01455.x

[ece32986-bib-0044] Guisande, C. , Granado‐Lorencio, C. A. , Toja‐Santillana, J. , León, L. , & León‐Muez, D. (2008). Identification of the main factors in structuring rotifer community assemblages in ponds of Doñana National Park using amino acid composition of the species. Limnetica, 28, 273–284.

[ece32986-bib-0045] Hairston, N. G. (1996). Zooplankton egg banks as biotic reservoirs in changing environments. Limnology and Oceanography, 41, 1087–1092.

[ece32986-bib-0046] Havel, E. , Eisenbacher, E. M. , & Black, A. A. (2000). Diversity of crustacean zooplankton in riparian wetlands: Colonization and egg banks. Aquatic Ecology, 34, 63–76.

[ece32986-bib-0047] Hebert, P. D. N. , Penton, E. , Burns, J. , et al. (2004). Ten species in one: DNA barcoding reveals cryptic species in the neotropical skipper butterfly *Astraptes fulgerator* . Proceedings of the National Academy of Sciences, 101, 14812–14817. 10.1073/pnas.0406166101 PMC522015 15465915

[ece32986-bib-0048] Hebert, P. D. N. , Ratnasingham, S. , & deWaard, J. R. (2003). Barcoding animal life: Cytochrome *c* oxidase subunit 1 divergences among closely related species. Proceedings of the Royal Society of London B: Biological Sciences, 270, S96–S99. 10.1098/rsbl.2003.0025 PMC1698023 12952648

[ece32986-bib-0049] Herzig, A. (1983). Comparative studies on the relationship between temperature and duration of embryonic development of rotifers. Hydrobiologia, 104, 237–246.

[ece32986-bib-0050] Kekkonen, M. , & Hebert, P. D. N. (2014). DNA barcode‐based delineation of putative species: Efficient start for taxonomic workflows. Molecular Ecology Resources, 14, 706–715. 24479435 10.1111/1755-0998.12233 PMC4264940

[ece32986-bib-0051] Koste, W. (1978). Rotatoria: Die Rädertiere Mitteleuropas (Überordnung Monogononta): Ein Bestimmungswerk begründet von Max Voigt, vol. 2. Stuttgart: Gebrüder Borntraeger, 673.

[ece32986-bib-0052] Leasi, F. , Tang, C. Q. , De Smet, W. H. , & Fontaneto, D. (2013). Cryptic diversity with wide salinity tolerance in the putative euryhaline *Testudinella clypeata* (Rotifera, Monogononta). Zoological Journal of the Linnean Society, 168, 17–28.

[ece32986-bib-0053] Li, L. , Niu, C. J. , & Ma, R. (2010). Rapid temporal succession identified by COI of the rotifer *Brachionus calyciflorus* Pallas in Xihai Pond, Beijing, China, in relation to ecological traits. Journal of Plankton Research, 32, 951–959.

[ece32986-bib-0054] López, T. , Toja, J. , & Gabellone, N. A. (1991). Limnological comparison of two peridunar ponds in the Doñana National Park (Spain). Archiv für Hydrobiologie, 120, 357–378.

[ece32986-bib-0055] Ludwig, W. , Strunk, O. , Westram, R. , Richter, L. , Meier, H. , Yadhukumar Buchner, A. , … Schleifer, K. H. (2004). ARB: A software environment for sequence data. Nucleic Acids Research, 32, 1363–1371. 14985472 10.1093/nar/gkh293 PMC390282

[ece32986-bib-0056] May, L. (1986). Rotifer sampling‐a complete species list from one visit. Hydrobiologia, 134, 117–120.

[ece32986-bib-0057] Mergeay, J. , Verschuren, D. , & De Meester, L. (2005). *Daphnia* species diversity in Kenya, and a key to the identification of their ephippia. Hydrobiologia, 542, 261–274.

[ece32986-bib-0058] Miller, M. A. , Pfeiffer, W. , & Schwartz, T. (2010). Creating the CIPRES Science Gateway for inference of large phylogenetic trees. *Proceedings of the Gateway Computing Environments Workshop (GCE)* New Orleans, LA, 1–8.

[ece32986-bib-0059] Mills, S. , Alcántara‐ Rodríguez, J. A. , Ciros‐Pérez, J. , Gómez, A. , Hagiwara, A. , Galindo, K. H. , … Walsh, E. (2016). Fifteen species in one: Deciphering the *Brachionus plicatilis* species complex (Rotifera, Monogononta) through DNA taxonomy. Hydrobiologia, 1–20. https://doi.org/10.1007/s10750-016-2725-7

[ece32986-bib-0060] Montero‐Pau, J. , Gómez, A. , & Muñoz, J. (2008). Application of an inexpensive and high‐throughput genomic DNA extraction method for the molecular ecology of zooplanktonic diapausing eggs. Limnology and Oceanography: Methods, 6, 218–222.

[ece32986-bib-0061] Montero‐Pau, J. , Ramos‐Rodríguez, E. , Serra, M. , & Gómez, A. (2011). Long‐term coexistence of rotifer species. PLoS ONE, 6, 1–9. 10.1371/journal.pone.0021530 PMC3125258 21738691

[ece32986-bib-0062] Moreno, E. , Pérez‐Martínez, C. & Conde‐Porcuna, J. M. (2016). Dispersal of zooplankton dormant propagules by wind and rain in two aquatic systems. Limnetica, 35, 323–336.

[ece32986-bib-0501] Moreno, E. , Pérez‐Martínez, C. , & Conde‐Porcuna, J. M. (2016). Zooplankton diversity and community assemblages in Mediterranean ponds with different hydroregimes. Manuscript in preparation.

[ece32986-bib-0063] Muñoz, J. (2010). Diversity and distribution of diapausing aquatic invertebrates in inland wetlands: An ecosystem conservation viewpoint. Journal for Nature Conservation, 18, 55–62.

[ece32986-bib-0064] Munuswamy, N. , Hagiwara, A. , Murugan, G. , Hirayama, K. , & Dumont, H. J. (1996). Structural differences between the resting eggs of *Brachionus plicatilis* and *Brachionus rotundiformis* (Rotifera, Brachionidae): An electron microscopic study. Hydrobiologia, 318, 219–223.

[ece32986-bib-0065] Myers, N. , Mittermeier, R. A. , Mittermeier, C. G. , Da Fonseca, G. A. B. , & Kent, J. (2000). Biodiversity hotspots for conservation priorities. Nature, 403, 853–858. 10706275 10.1038/35002501

[ece32986-bib-0066] Obertegger, U. , Flaim, G. , & Fontaneto, D. (2014). Cryptic diversity within the rotifer *Polyarthra dolichoptera* along an altitudinal gradient. Freshwater Biology, 59, 2413–2427.

[ece32986-bib-0067] Obertegger, U. , Fontaneto, D. , & Flaim, G. (2012). Using DNA taxonomy to investigate the ecological determinants of plankton diversity: Explaining the occurrence of *Synchaeta* spp. (Rotifera, Monogononta) in mountain lakes. Freshwater Biology, 57, 1545–1553.

[ece32986-bib-0068] Oertli, B. , Biggs, J. , Céréghino, R. , & Grillas, P. (2005). Conservation and monitoring of pond biodiversity: Introduction. Aquatic Conservation: Marine and Freshwater Ecosystems, 15, 535–540.

[ece32986-bib-0069] Ortells, R. , Gómez, A. , & Serra, M. (2003). Coexistence of cryptic rotifer species: Ecological and genetic characterisation of *Brachionus plicatilis* . Freshwater Biology, 48, 2194–2202.

[ece32986-bib-0070] Ortells, R. , Gómez, A. , & Serra, M. (2006). Effects of duration of the planktonic phase on rotifer genetic diversity. Archiv für Hydrobiologie, 167, 203–216.

[ece32986-bib-0071] Palazzo, F. , Bonecker, C. C. , & Fernandes, A. P. C. (2008). Resting cladoceran eggs and their contribution to zooplankton diversity in a lagoon of the Upper Paraná River floodplain. Lakes & Reservoirs: Research & Management, 13, 207–214.

[ece32986-bib-0072] Pérez‐Martínez, C. , Barea‐Arco, J. , Conde‐Porcuna, J. M. , & Morales‐Baquero, R. (2007). Reproduction strategies of *Daphnia pulicaria* population in a high mountain lake of Southern Spain. Hydrobiologia, 594, 75–82.

[ece32986-bib-0073] Piscia, R. , Guilizzoni, P. , Fontaneto, D. , Vignati, D. , Appleby, P. G. , & Manca, M. (2012). Dynamics of rotifer and cladoceran resting stages during copper pollution and recovery in a subalpine lake. Annales De Limnologie‐International Journal of Limnology, 48, 151–160.

[ece32986-bib-0074] Pons, J. , Barraclough, T. G. , Gómez‐Zurita, J. , Cardoso, A. , Duran, D. P. , Hazell, S. , … Vogler, A. P. (2006). Sequence based species delimitation for the DNA taxonomy of undescribed insects. Systematic Biology, 55, 595–609. 16967577 10.1080/10635150600852011

[ece32986-bib-0075] Pourriot, R. , & Snell, T. W. (1983). Resting eggs in rotifers. Hydrobiologia, 104, 213–224.

[ece32986-bib-0076] Puillandre, N. , Lambert, A. , Brouillet, S. , & Achaz, G. (2012). ABGD, automatic barcode gap discovery for primary species delimitation. Molecular Ecology, 21, 1864–1877. 21883587 10.1111/j.1365-294X.2011.05239.x

[ece32986-bib-0502] R Development Core Team (2015). R: A language and environment for statistical computing. Vienna, Austria: R Foundation for Statistical Computing ISBN 3‐900051‐07‐0. Retrieved from http://www.R-project.org

[ece32986-bib-0077] Rambaut, A. , Suchard, M. A. , Xie, D. , & Drummond, A. J. (2013). Tracer v1.5. Retrieved from http://beast.bio.ed.ac.uk/tracer

[ece32986-bib-0078] Ricci, C. (2001). Dormancy patterns in rotifers. Hydrobiologia, 446(447), 1–11.

[ece32986-bib-0079] Rojo, C. , Rodrigo, M. A. , & Barón‐Rodríguez, M. (2007). Dynamics of the planktonic food web in Colgada Lake (Lagunas de Ruidera Natural Park). Limnetica, 26, 251–264.

[ece32986-bib-0080] Rozas, J. , & Rozas, R. (1995). DnaSP, DNA sequence polymorphism: An interactive program for estimating population genetics parameters from DNA sequence data. Bioinformatics, 11, 621–625. 10.1093/bioinformatics/11.6.621 8808578

[ece32986-bib-0081] Sala, O. , Chapin, I. S. , Armesto, J. , Berlow, E. , Bloomfield, J. , Dirzo, R. , … Wall, D. H. (2000). Global biodiversity scenarios for the year 2100. Science, 287, 1770–1774. 10710299 10.1126/science.287.5459.1770

[ece32986-bib-0082] Schröder, T. (2005). Diapause in monogonont rotifers. Hydrobiologia, 546, 291–306.

[ece32986-bib-0083] Schröder, T. , & Walsh, E. J. (2007). Cryptic speciation in the cosmopolitan *Epiphanes senta* complex (Monogononta, Rotifera) with the description of new species. Hydrobiologia, 593, 129–140.

[ece32986-bib-0084] Schwartz, S. S. , & Hebert, P. D. N. (1987). Methods for the activation of the resting eggs of *Daphnia* . Freshwater Biology, 17, 373–379.

[ece32986-bib-0085] Segers, H. (2007). A global checklist of the rotifers (Phylum Rotifera). Zootaxa, 1564, 1–104.

[ece32986-bib-0503] Serra, M. , & King, C. E. (1999). Optimal rates of bisexual reproduction in cyclical parthenogens with density‐dependent growth. Journal of Evolutionary Biology, 12, 263–271.

[ece32986-bib-0086] Serrano, L. , & Fahd, K. (2005). Zooplankton communities across a hydroperiod gradient of temporary ponds in the Doñana National Park (SW Spain). Wetlands, 25, 101–111.

[ece32986-bib-0087] Serrano, L. , & Toja, J. (1998). Interannual variability in the zooplankton community of shallow temporary pond. Verhandlungen der Internationalen Vereinigung für Theoretische und Angewandte Limnologie, 26, 1575–1581.

[ece32986-bib-0088] Snell, T. W. , Burke, B. E. , & Messur, S. D. (1983). Size and distribution of resting eggs in a natural population of the rotifer *Brachionus plicatilis* . Gulf Research Reports, 7, 285–287.

[ece32986-bib-0089] Taberlet, P. , Coissac, E. , Pompanon, F. , Brochmann, C. , & Willerslev, E. (2012). Towards next‐generation biodiversity assessment using DNA metabarcoding. Molecular Ecology, 21, 2045–2050. 22486824 10.1111/j.1365-294X.2012.05470.x

[ece32986-bib-0090] Tamura, K. , Peterson, D. , Peterson, N. , Stecher, G. , Nei, M. , & Kumar, S. (2011). MEGA5: Molecular evolutionary genetics analysis using maximum likelihood, evolutionary distance, and maximum parsimony methods. Molecular Biology and Evolution, 28, 2731–2739. 21546353 10.1093/molbev/msr121 PMC3203626

[ece32986-bib-0091] Tang, C. Q. , Leasi, F. , Obertegger, U. , Kieneke, A. , Barraclough, T. G. , & Fontaneto, D. (2012). The widely used small subunit 18S rDNA molecule greatly underestimates true diversity in biodiversity surveys of the meiofauna. Proceedings of the National Academy of Sciences, 109, 16208–16212. 10.1073/pnas.1209160109 PMC3479535 22988084

[ece32986-bib-0092] Vandekerkhove, J. , Declerck, S. , Brendonck, L. , Conde‐Porcuna, J. M. , Jeppesen, E. , & De Meester, L. (2005). Hatching of cladoceran resting eggs: Temperature and photoperiod. Freshwater Biology, 50, 96–104.

[ece32986-bib-0093] Vandekerkhove, J. , Declerck, S. , Brendonck, L. , Conde‐Porcuna, J. M. , Jeppesen, E. , Johansson, L. S. , & De Meester, L. (2005). Uncovering hidden species: Hatching diapausing eggs for the analysis of cladoceran species richness. Limnology and Oceanography: Methods, 3, 399–407.

[ece32986-bib-0094] Vandekerkhove, J. , Declerck, S. , Jeppesen, E. , Conde‐Porcuna, J. M. , Brendonck, L. , & De Meester, L. (2005). Dormant propagule banks integrate spatio‐temporal heterogeneity in cladoceran communities. Oecologia, 142, 109–116. 15378346 10.1007/s00442-004-1711-3

[ece32986-bib-0095] Vandekerkhove, J. , Declerck, S. , Vanhove, M. , Brendonck, L. , Jeppesen, E. , Conde‐Porcuna, J. M. , & De Meester, L. (2004). Use of ephippial morphology to assess richness of anomopods: Potentials and pitfalls. Journal of Limnology, 63, 75–84.

[ece32986-bib-0096] Villesen, P. (2007). FaBox: An online toolbox for fasta sequences. Molecular Ecology Notes, 7, 965–968.

[ece32986-bib-0097] Wallace, R. L. (2002). Rotifers: Exquisite metazoans. Integrative and Comparative Biology, 42, 660–667. 21708762 10.1093/icb/42.3.660

[ece32986-bib-0098] Walsh, E. J. , May, L. , & Wallace, R. L. (2016). A metadata approach to documenting sex in phylum Rotifera: Diapausing embryos, males, and hatchlings from sediments. Hydrobiologia, 1–12.

[ece32986-bib-0099] Walsh, E. J. , Schröder, T. , Wallace, R. L. , & Rico‐Martínez, R. (2009). Speciation in *Lecane bulla* (Monogononta: Rotifera) in Chihuahuan Desert waters. Verhandlungen Internationale Vereinigung für Limnologie, 30, 1046–1050.

[ece32986-bib-0100] Xiang, X. L. , Xi, Y. L. , Wen, X. L. , Zhang, G. , Wang, J. X. , & Hu, K. (2011). Genetic differentiation and phylogeographical structure of the *Brachionus calyciflorus* complex in eastern China. Molecular Ecology, 20, 3027–3044. 21672065 10.1111/j.1365-294X.2011.05147.x

