# Supplementary material for: Barcoding rotifer biodiversity in Mediterranean ponds using diapausing egg banks
Source: Ecol Evol. 2017 May 27;7(13):4855–67. doi: 10.1002/ece3.2986 (PMC5496561; doi:10.1002/ece3.2986)
Supplement: Supplementary file 2 [file ECE3-7-4855-s002.pdf]

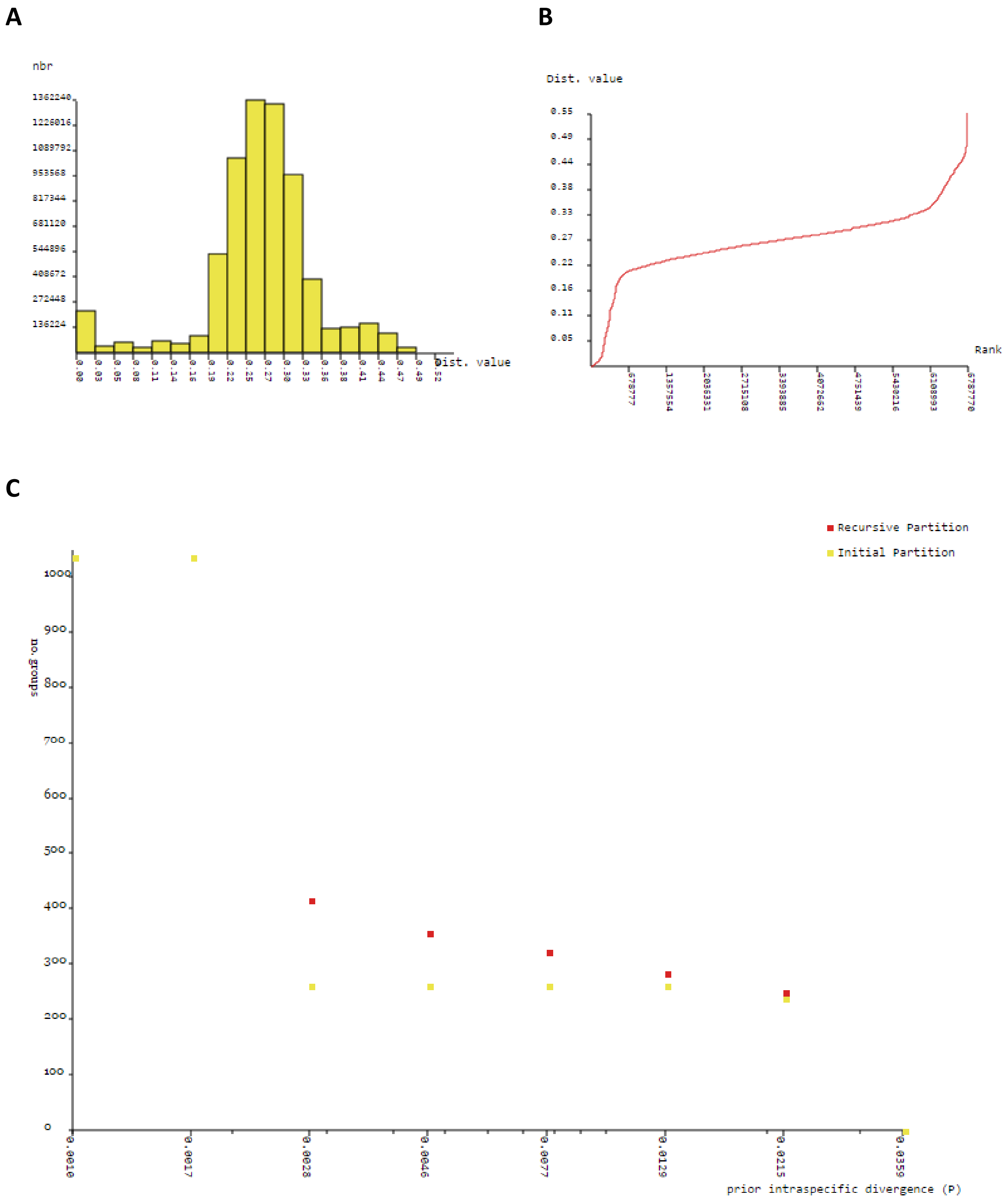

Figure S2. Barcode gap analysis generated by Automatic Barcode Gap Discovery (ABGD) (Puillandre et al. 2012). (A) histogram with distributions of Jukes-Cantor distances (Jukes & Cantor, 1969) between each pair of COI sequences, (B) ranked distances and (C) automatic partition based on Dataset2.
