# Supplementary figures and images for: Barcoding rotifer biodiversity in Mediterranean ponds using diapausing egg banks
Source: Ecol Evol. 2017 May 27;7(13):4855–67. doi: 10.1002/ece3.2986 (PMC5496561; doi:10.1002/ece3.2986)

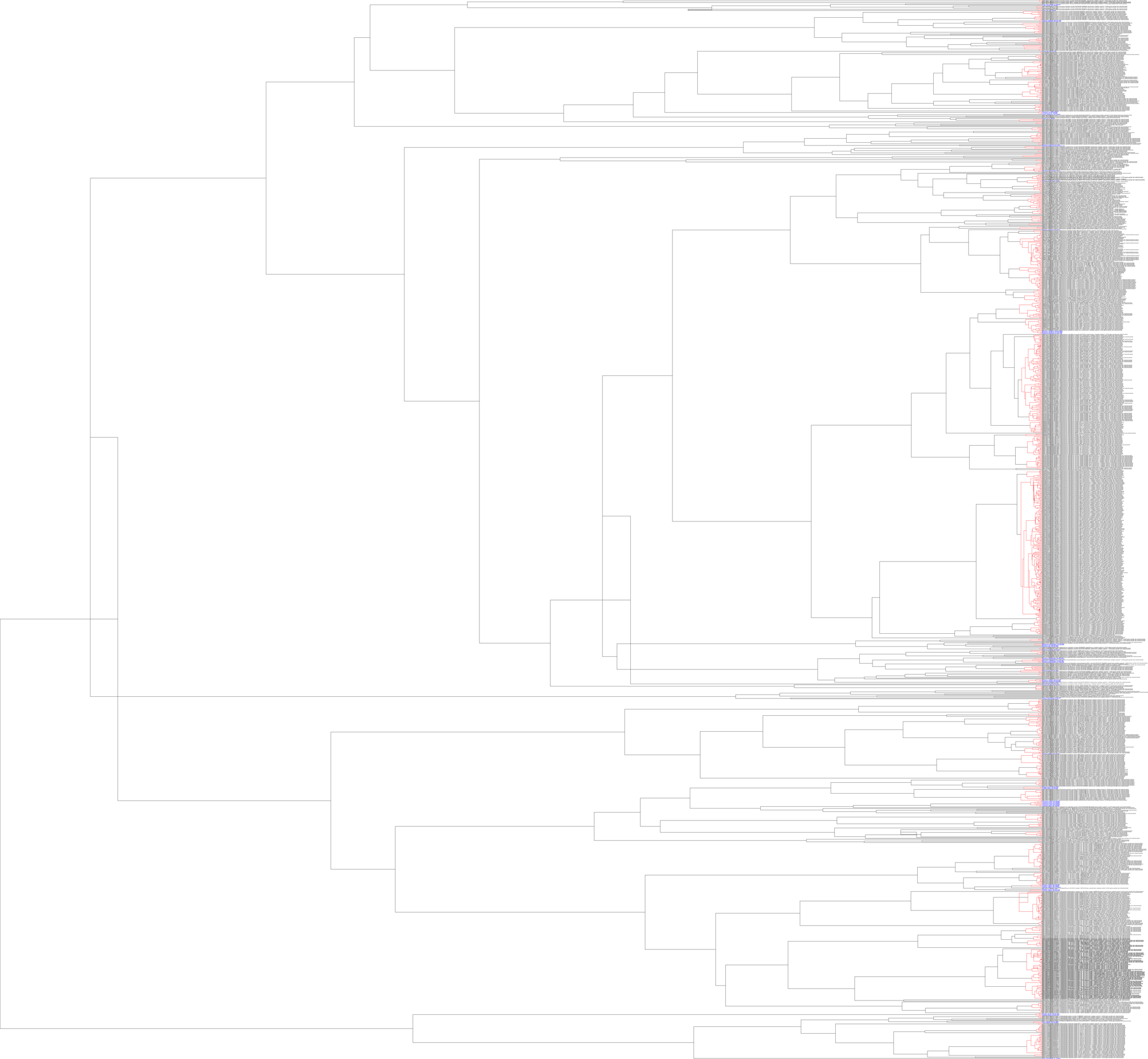

Supplement: Supplementary file 3 [file ECE3-7-4855-s003.pdf]
