# Supplementary material for: Barcoding rotifer biodiversity in Mediterranean ponds using diapausing egg banks
Source: Ecol Evol. 2017 May 27;7(13):4855–67. doi: 10.1002/ece3.2986 (PMC5496561; doi:10.1002/ece3.2986)
Supplement: Supplementary file 4 [file ECE3-7-4855-s004.docx]

**Table S1.** Uncorrected p-distances between each pair of GMYC entities considered as potential cryptic species, Jukes-Cantor model (JC) and Kimura’s two-parameter model (K2P)

|  | **P-distance** | **K2P** | **JC** |
| --- | --- | --- | --- |
| *Brachionus* Almenara - *Brachionus* Tiscar | 0.1600 ± 0.0167 | 0.1813 ± 0.0223 | 0.1800 ± 0.0212 |
| *B. quadridentatus* I - *B. quadridentatus* II | 0.1564 ± 0.0159 | 0.1771 ± 0.0205 | 0.1755 ± 0.0205 |
| *B. quadridentatus* I - *B. quadridentatus* III | 0.1621 ± 0.0166 | 0.1839 ± 0.0217 | 0.1826 ± 0.0212 |
| *B. quadridentatus* II - *B. quadridentatus* III | 0.1642 ± 0.0162 | 0.1866 ± 0.0214 | 0.1853 ± 0.0200 |
| *H. fennica* I - *Hexarthra fennica* II | 0.2431 ± 0,0195 | 0.3003 ± 0.0308 | 0.2939 ± 0.0286 |
| *K. tropica* I - *K. tropica* II | 0.0378 ± 0.0083 | 0.0392 ± 0.0094 | 0.0388 ± 0.0090 |
| *Polyarthra vulgarica* I - *Polyarthra vulgarica* II | 0.0449 ± 0.0089 | 0.0467 ± 0.0092 | 0.0463 ± 0.0092 |
| *Polyarthra vulgarica* I - *Polyarthra vulgarica* III | 0.1726 ± 0.0148 | 0.1805 ± 0.0208 | 0.1600 ± 0.0208 |
| *Polyarthra vulgarica* I - *Polyarthra vulgarica* IV | 0.1600 ± 0.0175 | 0.1973 ± 0.0224 | 0.1961 ± 0.0224 |
| *Polyarthra vulgarica* II - *Polyarthra vulgarica* III | 0.1726 ± 0.0171 | 0.1973 ± 0.0092 | 0.1961 ± 0.0216 |
| *Polyarthra vulgarica* II - *Polyarthra vulgarica* IV | 0.1782 ± 0.0175 | 0.2050 ± 0.0229 | 0.2035 ± 0.0228 |
| *Polyarthra vulgarica* III - *Polyarthra vulgarica* IV | 0.1284 ± 0.0148 | 0.1433 ± 0.0184 | 0.1408 ± 0.0176 |
