# Supplementary material for: Barcoding rotifer biodiversity in Mediterranean ponds using diapausing egg banks
Source: Ecol Evol. 2017 May 27;7(13):4855–67. doi: 10.1002/ece3.2986 (PMC5496561; doi:10.1002/ece3.2986)
Supplement: Supplementary file 5 [file ECE3-7-4855-s005.docx]

**Table S2.** Results of the Automatic Barcode Gap Discovery (ABGD) analyses for each substitution model, including the number of groups obtained in the initial and recursive partition in the different prior intraespecific divergences (*P*).

|  |  |  | **Prior intraspecific divergence (*P*)** | | | | | | | | | |  |
| --- | --- | --- | --- | --- | --- | --- | --- | --- | --- | --- | --- | --- | --- |
| **Substitution model** | **X** | **Partition** | **0.0028** | | **0.0046** | | **0.0077** | | **0.0129** | | **0.0215** | | **0.0359** |
|  |  |  | Dataset 1 | Dataset 2 | Dataset 1 | Dataset 2 | Dataset 1 | Dataset 2 | Dataset 1 | Dataset 2 | Dataset 1 | Dataset 2 | Dataset 1 |
| **JC69** | 1.5 | Initial | 34 | 263 | 34 | 263 | 34 | 263 | 34 | 263 | 34 | 263 | 34 |
|  |  | Recursive | 43 | 418 | 42 | 359 | 42 | 325 | 41 | 287 | 38 | 253 | 35 |
| **K80** | 1.5 | Initial | 34 | 263 | 34 | 263 | 34 | 263 | 34 | 263 | 34 | 263 | 34 |
|  |  | Recursive | 43 | 416 | 42 | 359 | 42 | 331 | 41 | 287 | 38 | 250 | 35 |
| **Simple distance** | 1.5 | Initial | 34 | 263 | 34 | 263 | 34 | 263 | 34 | 263 | 34 | 241 | 34 |
|  |  | Recursive | 40 | 309 | 40 | 290 | 38 | 282 | 38 | 267 | 38 | 249 | 35 |

**JC69**, Jukes-Cantor substitution model; **K80**, Kimura 2-parameter substitution model; **Simple distance**, p-distance. X, relative gap width
