# Supplementary material for: Barcoding rotifer biodiversity in Mediterranean ponds using diapausing egg banks
Source: Ecol Evol. 2017 May 27;7(13):4855–67. doi: 10.1002/ece3.2986 (PMC5496561; doi:10.1002/ece3.2986)
Supplement: Supplementary file 7 [file ECE3-7-4855-s007.docx]

#Dataset1 100,000,000; log 5,000; Number of trees or samples=10,000#

library(ape)

library(MASS)

library(paran)

library(splits)

getwd()

setwd("C:/Users/Usuario/Documents/Paper barcoding/Dataset1/Cipres_Dataset1_GMYC/Definitivo")

D1tree<-read.nexus("p.tree")

plot.phylo(D1tree)

ltt.plot(D1tree, log='y')

D1GMYC<-gmyc(D1tree, method = 'single', interval = c(0,5), quiet = FALSE)

pdf(file="D1GMYC.pdf", width=80, height=72)

plot(D1GMYC)

dev.off()

summary(D1GMYC)

Result of GMYC species delimitation

method: single

likelihood of null model: 251.9922

maximum likelihood of GMYC model: 293.8043

likelihood ratio: 83.62426

result of LR test: 0***

number of ML clusters: 16

confidence interval: 16-16

number of ML entities: 36

confidence interval: 34-38

threshold time: -0.02032557

#Dataset2 100,000,000; log 5,000; Number of trees or samples=10,000#

library(ape)

library(MASS)

library(paran)

library(splits)

getwd()

setwd("C:/Users/Usuario/Documents/Paper barcoding/Dataset1/Cipres_Dataset2_GMYC/Definitivo")

D1tree<-read.nexus("p.tree")

plot.phylo(D2tree)

ltt.plot(D2tree, log='y')

D1GMYC<-gmyc(D1tree, method = 'single', interval = c(0,5), quiet = FALSE)

pdf(file="D2GMYC.pdf", width=80, height=72)

plot(D2GMYC)

dev.off()

summary(D2GMYC)

Result of GMYC species delimitation

method: single

likelihood of null model: 11089.35

maximum likelihood of GMYC model: 11239.9

likelihood ratio: 301.1003

result of LR test: 0***

number of ML clusters: 148

confidence interval: 139-158

number of ML entities: 285

confidence interval: 271-306

threshold time: -0.03509926
